# Supplementary material for: Comparing R-Bendamustine vs. R-CHOP Plus Maintenance Therapy as First-Line Systemic Treatment in Follicular Lymphoma: A Multicenter Retrospective GELTAMO Study
Source: Cancers (Basel). 2024 Mar 26;16(7):1285. doi: 10.3390/cancers16071285 (PMC11010804; doi:10.3390/cancers16071285)

	GLOBAL GROUP (n=405)			R-CHOP (N=245)			R-BENDA (N=160)		
	Age ≤60 (n=208)	Age >60 (n=188)	p	Age ≤60 (n=138)	Age >60 (n=107)	P	Age ≤60 (n=70)	Age >60 (n=81)	P
**1ST line discontinuation:**	4 (2%)	8 (4%)	0.24	3 (2%)	1 (1%)	0.80	1 (1%)	7 (9%)	0.11
Neutropenia:			0.27			0.06			0.94
**No**	93 (47%)	71 (39%)		54 (41%)	28 (27%)		39 (57%)	43 (55%)	
**Grade 1-2**	31(16%)	34 (19%)		25 (19%)	26 (25%)		6 (9%)	8 (10%)	
**Grade 3-4**	74(37%)	78 (43%)		51 (39%)	51 (49%)		23 (34%)	27 (35%)	
Anemia:			**0.005**			**0.007**			**0.05**
**No**	**144(71%)**	**102 (55%)**		**82 (60%)**	**43 (41%)**		**62 (94%)**	**59 (75%)**	
**Grade 1-2**	**53 (26%)**	**73 (40%)**		**50 (37%)**	**54 (51%)**		**3 (4%)**	**19 (24%)**	
**Grade 3-4**	5 (2%)	9 (5%)		**4 (3%)**	**8 (8%)**		**1 (1%)**	**1 (1%)**	
Thrombocytopenia:			0.18			0.15			0.57
**No**	173(85%) 25	145 (78%)		115(85%)	81 (76%)		58 (87%)	64 (81%)	
**Grade 1-2**	(12%)	31 (17%)		18 (13%)	18 (17%)		7 (10%)	13 (16%)	
**Grade 3-4**	5 (2%)	9 (5%)		3 (2%)	7 (7%)		2 (3%)	2 (2%)	
Liver toxicity:			0.42			0.51			0.54
**No**	189(94%)	179 (97%)		125(93%)	101(95%)		64 (97%)	78 (99%)	
**Grade 1-2**	9 (4%)	4 (2%)		8 (6%)	3 (3%)		1 (1%)	1 (1%)	
**Grade 3-4**	3 (1%)	2 (1%)		2 (1%)	2 (2%)		1 (1%)	0 (0%)	
Renal toxicity:			0.09			0.66			0.086
**No**	198(98%)	181 (98%)		133(98%)	105(99%)		65 (97%)	76 (96%)	
**Grade 1-2**	1 (0.5%)	4 (2%)		1 (1%)	1 (1%)		0 (0%)	3 (4%)	
**Grade 3-4**	3 (1%)	0 (0%)		1 (1%)	0 (0%)		2 (3%)	0 (0%)	
Infections:	46 (23%)	45 (25%)	0.72	32 (24%)	29 (28%)	0.55	14 (21%)	16 (21%)	1
Dermatologic toxicity:	**23 (12%)**	**10 (6%)**	**0.045**	14 (11%)	6 (6%)	0.25	9 (13%)	4 (5%)	0.088
Hospitalization:	29 (14%)	40 (22%)	0.046	22 (16%)	26 (26%)	0.1	7 (11%)	14 (18%)	0.24
Maintenance (m.) discontinuation:	36 (18%)	37 (20%)	0.4	17 (12%)	13 (12%)	0.56	19 (28%)	24 (30%)	0.86
**Discontinuation cause:**			0.082			**0.011**			0.72
Patient/physician decision	11 (31%)	5 (13%)		**6 (35%)**	**1 (8%)**		5 (26%)	4 (17%)	
Toxicity	11 (31%)	20 (54%)		**0 (0%)**	**5 (38%)**		11 (58%)	15 (62%)	
Progression	14 (39%)	12 (32%)		**11(65%)**	**7 (54%)**		3 (16%)	5 (21%)	
**Neutropenia m.:**			**0.002**			0.25			0.12
No	162(85%)	122 (70%)		112(87%)	78 (80%)		50 (79%)	44 (58%)	
Grade 1-2	12 (6%)	29 (17%)		8 (6%)	12 (12%)		4 (6%)	17 (22%)	
Grade 3-4	**17 (9%)**	**22 (13%)**		8 (6%)	7 (7%)		9 (14%)	15 (20%)	
**Anemia m.:**			**<0.001**			**0.002**			**0.04**
No	181(95%)	137 (81%)		12 (94%)	76 (79%)		61 (95%)	61 (82%)	
Grade 1-2	**9 (5%)**	**31 (18%)**		**7 (5%)**	**19 (20%)**		2 (3%)	**12 (16%)**	
Grade 3-4	1 (0.5%)	2 (1%)		0 (0%)	1 (1%)		1 (2%)	1 (1%)	
**Thrombocytopenia m.:**			**0.001**			**0.003**			0.22
No	182(95%)	143 (93%)		121(94%)	78 (80%)		61 (95%)	65 (88%)	
Grade 1-2	**7 (4%)**	**24 (14%)**		**5 (4%)**	**16 (16%)**		2 (3%)	8 (11%)	
Grade 3-4	3 (2%)	5 (3%)		2 (2%)	4 (4%)		1 (2%)	1 (1%)	
**Severe Infections m.:**	28 (15%)	33 (19%)	0.26	14 (11%)	13 (13%)	0.68	14 (22%)	20 (27%)	0.56
**Hospitalization m.:**	6 (3%)	18 (10%)	0.006	3 (2%)	10 (10%)	0.018	3 (5%)	8 (10%)	0.35
**Second malignancies:**	11 (5%)	16 (8%)	0.33	6 (4%)	10 (9%)	0.28	5 (7%)	6 (7%)	0.25

Supplementary Table S1. Toxicity distribution by age with the different chemo regimens.

Supplementary Table S2. Impact of different variables on six years OS and PFS (univariate analysis)

	6y-PFS	P	6y- OS	P
**Time from biopsy to initial treatment**		0.87		0.28
**median:**	70% (64-77)		89% (84-93)	
- **0-0.93**	72% (65-79)		92% (88-96)	
- **>0.93**				
**Sex**		0.11		0.029
**Male**	68% (62-75)		87% (82-92)	
**Female**	73% (67-80)		95% (91-98)	
**Age (years)**		0.049		<0.001
**≤60**	75% (68-81)		96% (94-99)	
**>60**	67% (60-74)		84% (79-90)	
**Ann Arbor stage**		0.028		0.91
**II-II bulky**	90% (79-100)		93% (83-100)	
**III-IV**	71% (66-76)		91% (88-94)	
**B symptoms present**		0.35		0.11
**No**	71% (65-78)		91% (88-95)	
**Yes**	71% (62-79)		89% (84-95)	
**ECOG performance**		0.029		<0.001
**status**	72% (67-77)		91% (88-95)	
**0-1**	48% (23-73)		73% (55-95)	
**2-4**				
**Bone marrow**		0.1		0.023
**involvement**	76% (70-82)		87% (81-92)	
**No**	66% (58-73)		95% (91-98)	
**Yes**				
**FLIPI score**		0.003		0.049
**0-1**	83% (74-93)		95% (89-100)	
**2**	73% (65-81)		92% (86-99)	
**3-5**	65% (57-72)		87% (82-93)	
**Histological grade**		0.67		0.76
**1**	75% (68-83)		94% (89-98)	
**2**	69% (61-77)		89% (84-95)	
**3a**	69% (58-80)		90% (83-98)	
**Induction regimen**		0.046		0.49
**R-CHOP**	67% (61-73)		91% (87-94)	
**R-Bendamustine**	79% (72-86)		91% (86-96)	

Supplementary Table S3. Impact of different variables on six years OS and PFS (univariate analisys) in FL grade 3a

	6y-PFS	P	6y- OS	P
**Time from biopsy to initial treatment median:**		0.57		0.79
- **0-0.93**	71% (57-85)		90% (80-99)	
- **>0.93**	62% (41-82)		90% (77-100)	
**Sex**		0.92		0.49
**Male**	69% (53-84)		87% (76-99)	
**Female**	68% (52-85)		93% (84-100)	
**Age (years)**		0.55		0.083
**≤60**	68% (52-84)		97% (90-100)	
**>60**	69% (53-86)		84% (71-97)	
**Ann Arbor stage**		0.12		0.49
**II-II bulky**	100% (NA)		100% (NA)	
**III-IV**	69% (57-81)		93% (86-100)	
**B symptoms present**		0.038		0.02
**No**	78% (66-90)		97% (92-100)	
**Yes**	52% (27-77)		81% (62-100)	
**ECOG performance status**		0.79		0.058
**0-1**	69% (56-82)		94% (87-100)	
**2-4**	67% (13-100)		67% (13-100)	
**Bone marrow involvement**		0.008		0.47
**No**	78% (66-90)		88% (78-98)	
**Yes**	48% (26-70)		96% (87-100)	
**FLIPI score**		0.22		0.29
**0-1**	86% (69-100)		100% (NA)	
**2**	68% (48-88)		87% (70-100)	
**3-5**	56% (37-75)		85% (71-99)	
**Induction regimen**		0.73		0.87
**R-CHOP**	68% (56-81)		91% (83-98)	
**R-Bendamustine**	63% (24-100)		80% (45-100)	

Supplementary Figure S1. OS comparing R-CHOP vs R-Bendamustine

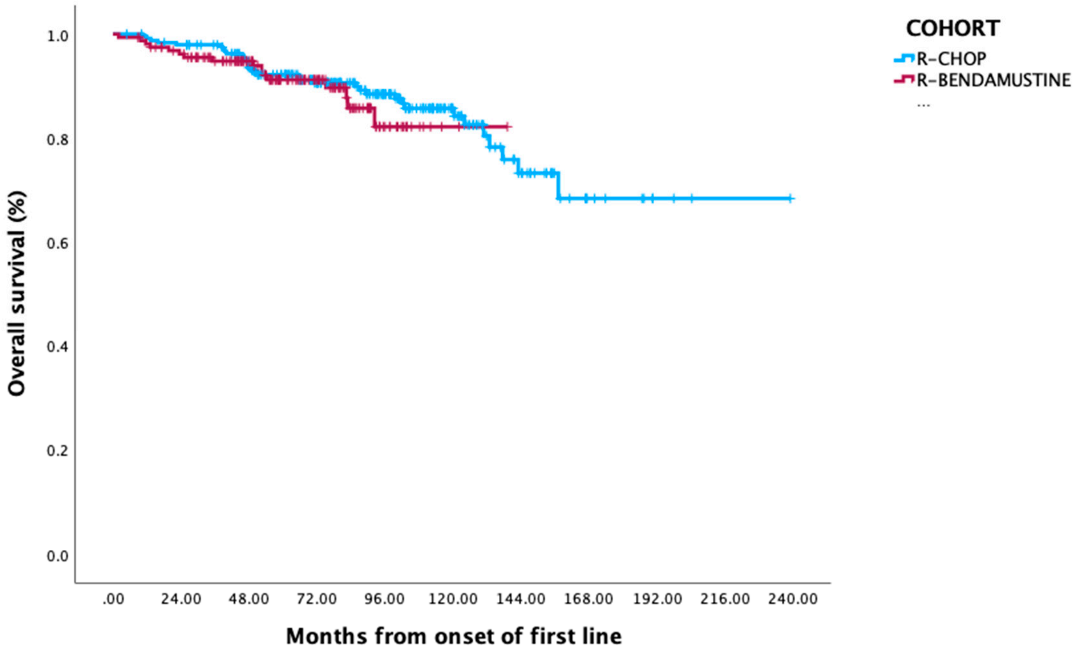

Supplement: Supplementary file 1 [file cancers-16-01285-s001.zip › cancers-2859477-supplementary.pdf]
